# Determination of chest x-ray cost using activity based costing approach at Penang General Hospital, Malaysia

**Published:** 2012-06-21

**Authors:** Muhammad Atif, Syed Azhar Syed Sulaiman, Asrul Akmal Shafie, Fahad Saleem, Nafees Ahmad

**Affiliations:** 1 Discipline of Clinical Pharmacy, School of Pharmaceutical Sciences, University Sains Malaysia, Penang, 11800, Malaysia; 2 School of Pharmaceutical Sciences, University Sains Malaysia, Penang, 11800, Malaysia; 3 Discipline of Social and Administrative Pharmacy, School of Pharmaceutical Sciences, University Sains Malaysia, Penang, 11800, Malaysia

**Keywords:** Activity based costing, Chest X-ray, radiology department, straight line depreciation

## Abstract

**Background:**

Activity based costing (ABC) is an approach to get insight of true costs and to solve accounting problems. It provides more accurate information on product cost than conventional accounting system. The purpose of this study was to identify detailed resource consumption for chest x-ray procedure.

**Methods:**

Human resource cost was calculated by multiplying the mean time spent by employees doing specific activity to their per-minute salaries. The costs of consumables and clinical equipments were obtained from the procurement section of the Radiology Department. The cost of the building was calculated by multiplying the area of space used by the chest X-ray facility with the unit cost of public building department. Moreover, straight-line deprecation with a discount rate of 3% was assumed for calculation of equivalent annual costs for building and machines. Cost of electricity was calculated by multiplying number of kilo watts used by electrical appliance in the year 2010 with electricity tariff for Malaysian commercial consumers (MYR 0.31 per kWh).

**Results:**

Five activities were identified which were required to develop one chest X-ray film. Human resource, capital, consumable and electricity cost was MYR 1.48, MYR 1.98, MYR 2.15 and MYR 0.04, respectively. Total cost of single chest X-ray was MYR 5.65 (USD 1.75).

**Conclusion:**

By applying ABC approach, we can have more detailed and precise estimate of cost for specific activity or service. Choice of repeating a chest X-ray can be based on our findings, when cost is a limiting factor.

## Background

Health care organizations use cost accounting to estimate unit cost of their services that could helps to plan a realistic budget and price for the service [1]. Conventional costing systems utilize a single, volume-based cost driver. In most cases this type of costing system allocates the overhead costs to products on the basis of their relative usage of direct labor. This method, has therefore, failed to cope with the challenges of rapidly evolving process and product technologies. It has been well established fact that conventional accounting method overestimates high volume products and underestimates low volume products. This gives an incorrect relationship between production and costs [2].

To date, most of hospital managers rely on information from conventional accounting system that was designed when competition was local rather than global and when pace and quality of item or service was less decisive for success [3]. However, many companies have found a better cost accounting method named as activity based costing (ABC) [4]. ABC approach allows an organization to utilize its resources in best possible way by providing insights into production process for delivering products or services to their consumers [5, 6]. In an activity based accounting system, cost of product or service is the sum of the costs of all the activities required to produce or deliver the service [7]. Accuracy of reported cost is directly proportional to number of activities studied and so does the cost of executing the study [7].

Chest X-rays are usually ordered by the doctors in patients complaining persistent cough, chest pain, hemoptysis, chest injury and short ness of breath. Chest X-rays may also be done if the patients are suspected of pulmonary tuberculosis, lung cancer or any other lung disease. In most of the chronic diseases like tuberculosis, lung cancer or chronic obstructive pulmonary disease, serial or repeated chest X-rays are ordered to monitor the progress of the disease [8].

Pneumonia, tuberculosis, lung cancer and lung diseases are leading causes of death in Malaysia [9], all of which require chest X-ray at some stage of disease management. Specially, in pulmonary tuberculosis, progress of the disease is routinely monitored by serial chest X-rays. Close contacts of pulmonary tuberculosis patients are also required to undergo at least one chest X-ray as a part of contact tracing procedure [10]. Health care in Malaysian government hospitals is given free of charge [11] therefore, every effort should be made to ensure adequate utilization of the resources.

This study was conducted at Radiology Department of Penang General Hospital, Penang, Malaysia to determine cost of developing one chest X-ray film by using the ABC approach. To date, no study from this region has reported the cost of chest X-ray by using activity based costing. Moreover, existing literature provides little information on the exact resource utilization for developing one chest X-ray film. Notwithstanding this, we also compared our cost with the one reported by conventional accounting method [12] in the same setting. Findings of our study will enable policymakers and departmental managers to come up with an accurate financial allocation for chest X-ray facility. Our findings would also enable medical practitioners in their decision to order repeated chest X-rays of a patient, when cost is a limiting factor.

## Methods

Radiology Department of Penang General Hospital has a designated facility for chest X-ray (labeled as room 2). Material consisted of 31431 radiological examinations performed during the period of January 1 to December 31, 2010. All costs were calculated in Malaysian Ringgit (MYR) and converted to American dollars (USD) according to the 2011 exchange rate of USD1 = MYR3.19. Total costs were broken down as follows: human resource cost, capital cost, consumable cost and overhead cost.

### Human resource cost

An interview with key radiology personnel was conducted to identify principal activities for chest X-ray. This was followed by determination of the time taken to complete each activity by using a stop watch [13]. The duration was captured 15 times each for alternate three days and summarized as the mean, median, the 25th and 75th quartiles for each activity [13]. The personnel time for each employees involved was valued according to the pay scale of the Federal Civil Services Officers under the System of Remuneration Malaysia [14]. Prior to the valuation, these salaries were converted into the salary per minute (MYR/min) by assuming a daily working time of 8 hours and a monthly working time of 20 days. Cost of each employee per single activity was obtained by multiplying the mean time (minutes) spent by that employee doing a specific activity by his/her salary per minute (MYR/min). Finally, the total manpower cost incurred per service (i.e. X-ray) was the sum of human resource costs of all activities involved producing the service.

Moreover, human resource idle time cost was calculated by multiplying mean idle time between two consecutive activities of each employee with their salary per minute. However, this cost was not included in final cost.

### Capital costs (machines and building)

The costs of equipments were obtained from the procurement section of radiology department. The costs of the building were calculated by multiplying the area size for the service with the unit cost of public building (MYR 85/ft2). Area size of the chest X-ray facility was also provided by the public building department of Penang General Hospital. The useful life was assumed to be five years for clinical equipment and 30 years for building [15]. Moreover, straight-line deprecation with a discount rate of 3% was assumed. At the end of the asset's useful life, the resale value was considered to be 10% of the initial costs [16]. The equivalent annual cost for each was calculated based on the following equations:

**Resale value** = Asset cost × 0.1

**Present value** = Resale value × Discount rate

**Net present value of the asset cost** = Asset cost – Present value

**Equivalent annual cost** = Net present value of the asset cost / Annuity factor

The unit asset cost was obtained by dividing equivalent annual cost of each asset by the total number of X-ray films in the year 2010.

### Consumable costs

Consumables included X-ray film, fixer and developer reagents and envelop for developed film. The quantity and cost of each X-ray film and envelop was obtained from the procurement section of radiology department. Total cost of fixer and developer reagent per service was obtained by dividing total cost of reagents in one year divided by number of tests in year 2010.

### Electricity costs

Annual electric power consumption (kW/h) for X-ray machine, day light developer machine and tubes was calculated separately and then multiplied by unit price of one kW/h (MYR 0.312 /kWh) [17] to get annual electric cost for each electrical appliance. Annual electric cost for each appliance was divided by number of X-rays done in 2010 to get cost per X-ray.

### Ethical approval

Ethical approval was taken from Ministry of Health, Malaysia (ref. dim. KKM/NIHSEC/08/08/04P10-69).

## Results

### Human resource costs

Five distinct activities to produce an X-ray film were identified which include receiving and allocating specific number to patient (attendant 1), registering patient in log book (clerk), preparing & exposing patient to X-rays and developing film in day light machine (radiographer 1), labeling film envelop and validating/sorting films to meet standard criteria (radiographer 2) and dispatching films to respective wards/clinics (attendant 2). Radiographer 1 and 2 are the designated staff for chest X-ray, while clerk, attendant1 and attendant 2 are the shared human resources. Table 1 shows the details of human resource cost. Total human resource cost was MYR 1.48 (USD 0.46) excluding idle time cost. Idle time cost for radiographer 2 was highest among all the staff involved in chest X-ray.

**Table 1:** Human resource cost per chest X-ray film

Staff	Activities	Mean time (minutes)	Median time (25th,75th) (minutes)	Salary per minute (MYR)	Cost per unit (MYR)	Percentage from total cost	Idle time (seconds)	Idle time cost (MYR)
Attendant 1	Receiving and allocating number	1.15	0.98 (0.89,1.3)	0.141	0.162	10.9	8	0.018
Clerk	Patient registration	0.74	0.72 (0.55,1.0)	0.145	0.107	7.2	5	0.012
Radiographer 1	Preparing and exposing patient	3.43	3.0 (2.3,4.6)	0.211	0.723	48.7	12	0.042
Radiographer 2	Labeling and validating film	1.79	1.4 (1.2,2.0)	0.169	0.302	20.4	107	0.299
Attendant 2	Dispatching films	1.36	1.3 (1.3,1.4)	0.141	0.190	12.8	6	0.138
Human resource cost per chest X-ray					1.48	Idle time cost per chest X -ray		0.51

MYR: Malaysian Ringgit

### Capital costs

Capital costs included cost of X-ray machine (Philips™), cost of daylight developer equipment (Agfa, Compact EOS™) and cost of building (Room number 2). Cost (per film) of X-ray equipment was highest (MYR 1.629) followed by daylight developer equipment (MYR 0.318) and building (MYR 0.032). Total capital cost per chest X-ray film was MYR 1.979 (USD 0.62). Table 2 shows equivalent annual costs and unit costs of assets.

**Table 2:** Equivalent annual cost and unit cost of assets

Asset	Equivalent annual cost (MYR)	No of test done in 2010	Unit cost (MYR)	Percentage
X-ray machine	51208.3	31431	1.629	82.3
Daylight developer	44825.5	140973	0.318	16.1
Building	1010.4	31431	0.032	1.6
**Total unit asset cost**			**1.979**	**100**

MYR: Malaysian Ringgit

### Consumable costs

Cost of reagents (developer and fixer) and envelop per X-ray film was MYR 0.244 and MYR 0.257, respectively. X-ray film was the most costly item (MYR 1.652) among consumables. Total consumable cost per chest X-ray film was MYR 2.15 (USD 0.66).

### Overhead costs (electricity)

Electricity cost for X-ray equipment, daylight developer equipment and tubes was MYR 0.002, MYR 0.031 and MYR 0.01, respectively. Total electricity cost per chest X-ray films was MYR 0.043 (USD 0.01).

### Total cost per X-ray film

Total cost of one chest X-ray film was MYR 5.65 (USD 1.75) (Table 3).

**Table 3:** Overall cost per one chest X-ray film

Resources	Cost (MYR)	Cost (USD)	Percentage (%)
Human resources cost	1.48	0.46	26.3
Capital costs	1.98	0.62	35.4
Consumable costs	2.15	0.66	37.7
Overhead costs	0.04	0.01	0.57
**Total cost**	**5.65**	**1.75**	**100**

**MYR:** Malaysian Ringgit

## Discussion

Although, ABC has been successfully implemented in various manufacturing and service organizations, there have been only few reports on implementation of ABC in health care [18]. Current study aims to calculate the cost of chest X-ray using ABC approach at radiology department of Penang General Hospital, Penang, Malaysia. Among the human resource cost, radiographer 1 and 2 shared 48.8% and 20.4% of total human resource cost, respectively. Scheduling and registration (attendant 1 and clerk) cost was 18.1% which is almost similar to what reported in another study [18]. Idle time cost for radiographer 2 was around seven times higher (MYR 0.29 vs MYR 0.042) than radiographer 1. Moreover, radiographer 2 remained idle for around 1.78 minutes (107 seconds) which was almost equal to his activity time (1.79 minutes). This clearly suggests that he (radiographer 2) can share another similar activity in the radiology department, thus saving human resource cost.

X-ray machine shared 82.3% of per unit capital cost whereby daylight developer and building shared 16.2% and 1.6%, respectively. Among the consumables, X-ray film was the most costly item and shared 76.8% of total consumable cost.

Total cost of single chest X-ray was MYR 5.65 (USD 1.75) whereby consumables and capital cost shared 37.7% and 35.4%, respectively. In 2003, Elfatih et al [12] estimated the cost of chest X-ray in the same setting. In that study, cost of machine and building were excluded. Moreover, they provided very little or no information on the method for human resource and electricity cost calculation. According to their findings, cost of single chest X-ray was USD 4.86 which is 2.86 times higher than reported by our study. One of the possible reasons for this difference might be different cost accounting methods utilized in both studies.

This study is first of its kind in Malaysia and neighboring regions that has contributed significantly to policymakers and radiology departmental heads for adequate resource allocation for chest X-ray facility. Option of repeating a chest X-ray for a patient by the physician can be based on our findings, when cost is a limiting factor.

## Conclusion

By applying ABC approach, we can have more detailed and precise estimate of cost for specific activity or service. Process improvement and corrective actions can be made by properly identifying cost drivers such as time.
